# HCN Channels: New Therapeutic Targets for Pain Treatment

**DOI:** 10.3390/molecules23092094

**Published:** 2018-08-21

**Authors:** David Ramírez, Rafael Zúñiga, Guierdy Concha, Leandro Zúñiga

**Affiliations:** 1 Instituto de Ciencias Biomédicas, Universidad Autónoma de Chile, 5 Poniente 1670, Talca 3460000, Chile; damach.david@gmail.com; 2 Centro de Investigaciones Médicas, Programa de Investigación Asociativa en Cáncer Gástrico, Escuela de Medicina, Universidad de Talca, Talca 3460000, Chile; rafaelzunigah@gmail.com (R.Z.); guierdy@gmail.com (G.C.)

**Keywords:** HCN channels, HCN structure, HCN blockers, HCN channels expression, pain condition

## Abstract

Hyperpolarization-activated cyclic nucleotide-gated (HCN) channels are highly regulated proteins which respond to different cellular stimuli. The HCN currents (I_h_) mediated by HCN1 and HCN2 drive the repetitive firing in nociceptive neurons. The role of HCN channels in pain has been widely investigated as targets for the development of new therapeutic drugs, but the comprehensive design of HCN channel modulators has been restricted due to the lack of crystallographic data. The three-dimensional structure of the human HCN1 channel was recently reported, opening new possibilities for the rational design of highly-selective HCN modulators. In this review, we discuss the structural and functional properties of HCN channels, their pharmacological inhibitors, and the potential strategies for designing new drugs to block the HCN channel function associated with pain perception.

## 1. Introduction

### HCN Channels Structure and Function

**Figure 1:**
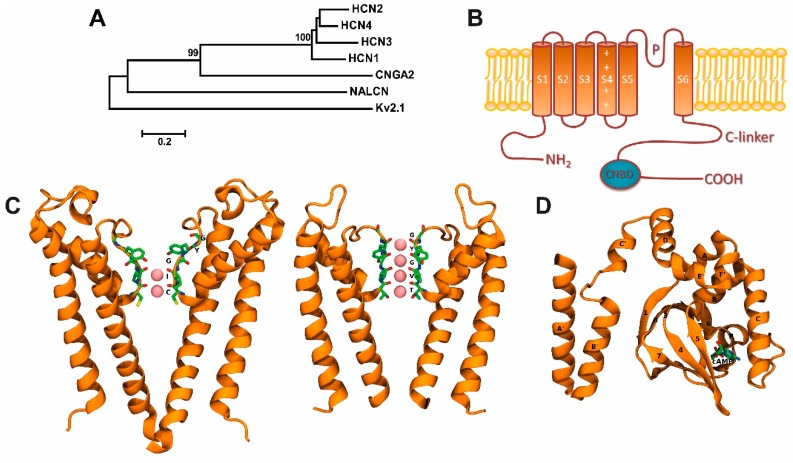
Hyperpolarization-activated cyclic nucleotide-gated (HCN) channels and their two- and three-dimensional structures: (**A**) Phylogenetic tree showing proteins in the human HCN channel family. It includes selected ion channels of Kv (voltage-gated K^+^ channel), NALCN (sodium leak channel, non-selective), and CNG (cyclic nucleotide-gated ion channel) families. Phylogenetic analysis was carried out with Molecular Evolutionary Genetics Analysis version 5 (MEGA5) software (www.megasoftware.net.) free of charge. Lines length, scaled below the tree, indicate the relative distance between nodes. Numbers on branches indicate bootstrap values (as a percentage). (**B**) Topological model proposed for HCN channels. Each subunit has one pore forming domain (P-loops) and six transmembrane domains (denoted S1–S6). The C-terminus of each subunit contains a cyclic nucleotide-binding domain (CNBD) connected to the sixth transmembrane α-heli x (S6) via the C-linker. (**C**) Left, HCN filter structure (Protein Data Bank, PDB: 5U6O [4]) in a ribbon representation, showing a weak K^+^-selective filter—K^+^ ion occupancy: 3 and 4 sites-. Right, KcsA filter structure (PDB:1K4C), showing a K^+^ selective filter—K^+^ ion occupancy: 1 to 4 sites-. The K^+^ ions in both filters, they are represented as pink spheres. A view of the K^+^ selectivity filter structure is shown on the right (**D**) cAMP-bound CNBD structure view (PDB:1Q5O [32]) in ribbon representation showing a cAMP molecule in a stick representation. Bothe C and D were prepared using PyMOL software version 2.0 (Schrödinger, LLC. New York, NY, USA).

Hyperpolarization-activated cyclic nucleotide-gated (HCN) channels are members of the voltage-gated pore loop channel superfamily [1,2,3], and are also related to the cyclic nucleotide-gated (CNG) channels as well as to the voltage-dependent K_V_10–K_V_12 channels [4] (Figure 1A). Besides the voltage-dependent gating, HCN channels are activated by intracellular cyclic nucleotides [5,6], including guanosine-3′,5′-cyclic monophosphate (cGMP) and adenosine-3′,5′-cyclic monophosphate (cAMP), while the modulation of I_h_ is similar for both cyclic nucleotides, with the same efficacy at least in mammalians, the apparent affinities of I_h_ are 10–100 fold higher for cAMP than for cGMP [7]. Hyperpolarization-activated cyclic nucleotide-gated channels are integrated by four subunits that together form a central pore. Each subunit contains a voltage-sensor domain and a pore domain contributing to the central pore [8]. However, this cyclic nucleotide modulatory effect depends on each HCN subunit [9,10], with the cAMP sensitivity higher for HCN2 and HCN4, weaker in HCN1, and absent in HCN3 [11,12]. The cGMP has a similar efficacy to cAMP, but with a lower apparent affinity [13].

The cAMP modulation, in HCN channels, is generated by a direct binding to the intracellular cyclic nucleotide binding domain (CNBD) located at C-terminal. This binding leads to accelerated activation kinetics and to a shift of the conductance voltage curve toward positive voltages (up to 20 mV) [1,2,3,5]. Additionally, the open probability (Po) of HCN channels can be increased by the cAMP binding, but unlike CNG channels, the cyclic nucleotides are not a prerequisite for channel opening [4]. At strong hyperpolarization, two occupied binding sites with cAMP are sufficient to generate the maximum Po [8,14,15], and at least two liganded subunits in trans positions are required to maintain the activation [8].

Moreover, in HCN channels the voltage dependence goes in opposite directions to the classical voltage-dependent ion channels, which opens with a depolarized stimulus. Hyperpolarization-activated cyclic nucleotide-gated channels are closed to a depolarized stimulus and opened to the membrane hyperpolarization [1,2,3].

In mammals, four HCN isoforms have been identified to encode for the subunits HCN1 to HCN4 [8]. To form a functional channel, HCN subunits (HCN1–4) need to assemble as tetramers. The HCN channels are able to form homo- or heterotetrameric complexes, generating channel subtypes with distinct biophysical properties [16]. Thus, each HCN subunit can be self-assembled in a homomeric architecture, and, excluding HCN2 and HCN3, all dual combinations of HCN subunits co-assemble to form functional heteromeric channels [17]; each subunit comprises six critical transmembrane domains (S1–S6), as well as an intracellular C- and N-terminal (Figure 1B). Similar to other ion channels from the voltage-gated family, the S4 transmembrane domain contains the voltage sensor and comprises several charged amino acids (Arg and Lys residues) [18,19,20,21].

Hyperpolarization-activated cyclic nucleotide-gated channels contain a GYG (Glycine-Tyrosine-Glycine) motif that confers the high selectivity for K^+^ ions observed in K^+^ channels [22]. However, the Na^+^/K^+^ permeability ratio in HCN channels is ~1/4 and also displays a small but significant permeability to Ca^2+^ ions [23]. For instance, at 2.5 mM of external Ca^2+^, the Ca^2+^ current in the native HCN current (I_h_) as well as in the expression system, where channels HCN2 and HCN4 are expressed, is about 0.5% [23,24]. This Ca^2+^ current of HCN channels is relatively small (0.47% in HCN2 and 0.6% in HCN4 channels) compared with the fractional Ca^2+^ currents in other ion channels, such as the nicotinic acetylcholine receptor (2.5%) [25], glutamate receptor (10% for NMDA, *N*-methyl-d-aspartic acid) [26], AMPA, α-amino-3-hydroxy-5-methyl-4-isoxazolepropionic acid/kainate receptors (0.5–5%) [27], CNG channels (10–80%) [28], and calcium channels (100%) [29]. However, this Ca^2+^ current through HCN channels may be enough to modulate Ca^2+^-dependent cellular functions [23,24]. Lee and MacKinnon [4] recently resolved, by cryoelectron microscopy, the three-dimensional structure of the human HCN1 subunit with (Protein Data Bank [PDB] ID: 5U6O) and without (PDB ID: 5U6P) its C-terminus. They reported that the selectivity filter, which contains only two out of four K^+^ binding sites (denoted as 1–4 from the extracellular to the intracellular side), adopts a non-canonical configuration [4], where the K^+^ ion occupies the 3 and 4 sites (Figure 1C, left). This explanation is the main difference between a weak K^+^-selective filter (HCN1 channel) and a K^+^-selective filter (e.g., KscA channel) where all sites are occupied by K^+^ (Figure 1C, right). These data may also explain why HCN channels are permeable to different ions, despite the presence of the GYG motif. In the pore of K^+^ selective channels, the four K^+^ binding sites are created by four layers of carbonyl oxygen atoms and one layer of threonine hydroxyl oxygen atoms (Figure 1C, right panel).

A cyclic nucleotide-binding domain (CNBD) is found at the intracellular C-terminus of HCN channels. The CNBD, connected to the S6 transmembrane domain by a C-linker, regulates the HCN channel activity by facilitating the cyclic adenosine monophosphate (cAMP) binding [30,31] (Figure 1B,D) promoting voltage-dependent activation of HCN channels [5] (Figure 1D). As indicated previously, HCN subunits present different sensitivity to cAMP. Both HCN2 and HCN4 exhibit high voltage dependence and kinetics of activation to cAMP, which is extremely weak in HCN1 and absent in HCN3 [11,12,32]. The CNBD three-dimensional structure in the presence and absence of cAMP has been solved by crystallography [32] and cryoelectron microscopy [4], and entails six alpha-helices (A–F) and one beta-roll in between the A and B helices [32,33,34,35,36,37] (Figure 1D). Lee and MacKinnon [4] demonstrated that cAMP binding in the absence of hyperpolarization is insufficient for channel opening; however, the cAMP binding induces structural changes, such as the rotation of the inner gate, which may facilitate its opening by changes in the membrane potential.

The HCN channels are widely expressed in peripheral sensory neurons, neurons in the central nervous system [38], and cardiac tissues [2,3,39,40,41]. The HCN channels generate inward current (I_h_)—a Na^+^/K^+^ current when the membrane potential is hyperpolarized, producing rhythmic electrical activity in specialized neurons of the brain [39,41,42] and in cardiac sinoatrial node cells [43]. Diverse functions have been attributed to I_h_ currents, including the determination of resting membrane potential (RMP), action potential (AP) firing rate, dendritic integration, and synaptic transmission [44].

Hyperpolarization-activated cyclic nucleotide-gated channel activity plays important roles in behavior and physiological process such as sleep and arousal, learning and memory, and anesthesia [38,45,46,47]. Misregulation of HCN channel activity has been shown to contribute to neurological and psychological disorders including pain, epilepsy, addiction, and anxiety [48,49,50,51,52].

## 2. HCN Channel Regulation

The cyclic nucleotide adenosine-3′,5′-cyclic monophosphate regulates the voltage dependence of HCN subunits [5], and promotes a shift of 10–25 mV in V0.5 in the HCN2 and HCN4 channels [7,10]. In contrast, the V0.5 displayed by HCN1 is only slightly shifted (shift by around 2–7 mV) [7,10], and HCN3 is not modulated by cyclic nucleotides [7,53].

In addition to cAMP, HCN channels are also allosterically regulated by other molecules, such as phosphatidylinositol 4,5-biphosphate, cholesterol, H^+^, and Cl^−^ ions [31], and modulated by several post-translational modifications, such as phosphorylation (e.g., Src, mitogen-activated protein serine/threonine kinase [p38-MAPK], protein kinase C [PKC], and Ca^2+^/calmodulin-dependent protein kinase II) [54,55,56,57]. For instance, Src shifts the HCN channels voltage dependence, thus accelerating their kinetics [58,59]. Also, p38-MAPK activates HCN channels by shifting the voltage dependence and depolarizing the membrane potential. In addition, PKC inactivates HCN channels. For example, PKC activation by phorbol 12,13-diacetate or 4β-phorbol 12-myristate 13-acetate downregulates HCN channels by shifting the voltage dependence to a more negative potential [60,61,62]. Nowadays, it is not clear how p38-MAPK and PKC modulate HCN channels.

As previously mentioned, cholesterol within lipid rafts can disrupt the HCN channel function since the HCN1, HCN2, and HCN4 subunits expression were downregulated after cholesterol depletion [63].

## 3. HCN Channels in the Central Nervous System

All HCN members (HCN1–4) have been found in central and peripheral nervous systems, where they are associated with synaptic integration, neuronal excitability, and the formation of resting membrane potentials [46]. Neurons highly express HCN channels [44]. In these excitable cells, HCN currents are stated as hyperpolarization currents (I_h_) and can inhibit the passive propagation of excitatory post-synaptic potentials [64,65,66,67]. On the other hand, HCN currents are defined as “funny” (I_f_) or pacemaker currents in cardiac cells [30,41,68,69]. Numerous studies have shown that HCN channels display high mRNA and protein expression levels in central nervous and cardiovascular systems [39,40,41,42,68]. For instance, in situ hybridization studies revealed high expression levels of HCN1 subunit in mice’s olfactory bulb, cerebral cortex, hippocampus, superior colliculus, and cerebellum [40,42]. The HCN1 subunit expression has also been detected at post-synaptic sites in the basket and Purkinje cells in rats’ cerebral cortex [70]. In the mouse brain, the HCN2 subunit is ubiquitously expressed with high expression levels in the olfactory bulb, hippocampus, thalamus, and brain stem [40,42]. In contrast, HCN3 subunit expression levels are very low in the brain [40], whereas the HCN4 subunit is only expressed in the thalamus and the olfactory bulb [40,42].

Immunochemistry studies have shown the localization of various HCN channels in different tissues [39,71]. The HCN1 subunit displays a cortical expression pattern with high expression levels in rats’ neocortex, hippocampus, superior colliculus, and cerebellum [39,71], while the HCN2 subunit shows ubiquitous expression in the brain in agreement with Moosmang and Santoro’s results [39,71]. On the other hand, the hypothalamus and thalamus are immunoreactive for HCN3 and HCN4 subunits, respectively [39], and these subunits are also expressed throughout the cerebellum [71]. Lastly, apical dendrites of hippocampal neurons are immunoreactive for various HCN channels [64,65,66,67].

Although the hyperpolarization of the membrane potential activates HCN channels expressed in the central nervous system tissues, a few channels are activated by resting membrane potentials (between −50 and −60 mV). The activation of HCN channels, by resting membrane potentials, brings them closer to the threshold which regulates the action potential [44,72]. Given the importance of HCN channels in excitable cells [44], further studies are needed to understand their regulation as well as the neuropathologies associated with its dysfunction (e.g., epilepsy).

## 4. The Role of HCN Channels in Pain Perception

Pain affects millions of individuals worldwide [73]. Chronic pain, which is often caused by several and different diseases, affects ~20% of the adult European population [74], turning it into a co-morbidity factor [75]. There is compelling evidence that pain is strongly related to ion channel regulation and/or modulation associated to neuronal activity in the peripheral nervous system [76]. Hyperpolarization-activated cyclic nucleotide-gated channels have been reported to participate in the pain process [31,77,78,79,80,81,82], where I_h_ can induce the repetitive firing of primary nociceptive neurons [83,84,85]. It is not fully clear the specific role of each HCN channel in pain perception. For instance, somatosensory afferent neurons highly express HCN1 and HCN2 subunits [41,77,83], although nociceptors (i.e., small somatosensory neurons) do not express the HCN1 subunit [85]. In contrast, nociceptors highly express the HCN2 subunit, which drives the firing rate during neuropathic and inflammatory-induced pain [79,81,82]. In agreement with HCN2 expression in the nociceptors, the HCN2 knockout mice did not show increased sensitivity to pain (i.e., hyperalgesia) in response to mechanical or thermal stimuli [79]. Additionally, neuropathic and inflammatory-induced pain is not attenuated in HCN1 knockout mice [85].

Diabetic patients develop a painful diabetic neuropathy (PDN)—a chronic pain condition induced by nerve damage [86]. The molecular mechanism of chronic pain in diabetes is poorly understood, and even at present there are no effective treatments [87]. Tsantoulas and colleagues [86] investigated the role of HCN2 as drivers of diabetic pain using mouse models (for diabetes type 1 and 2). The HCN2 channel activity blockade in small nociceptive neurons suppressed the diabetes-associated allodynia and prevented the nociceptive pathway in the spinal cord in mice [86]. Also, the pharmacological blockade with ivabradine, an HCN inhibitor, reduced chronic pain in mice with diabetes. These results suggest that selective HCN2 inhibitors might be a valuable treatment strategy for diabetic neuropathies [86]. In addition, Tsantoulas and colleagues [86] found that intracellular cAMP is increased in somatosensory neurons in an animal model of painful diabetes. This increased intracellular cAMP drives the diabetes-associated pain by facilitating the HCN2 activation with the consequent promotion of firing in primary nociceptive nerve fibers [86].

Even though distinct chemotherapeutic drugs have different modes of action, several drugs can also cause chronic pain. Three of this main chemotherapeutic classes, taxanes, platinum-based agents, and vinca alkaloids are commonly associated with chemotherapy-induced peripheral neuropathy (CIPN) [88]. Although antineoplastic drugs have a different mechanism of action and a dose/rate of CIPN occurrence, one common mechanism to these antineoplastic drugs is the change in the ion channel expression for primary afferent sensory neurons [88]. Oxaliplatin can induce peripheral neuropathy with hypersensitivity to mechanical and cold stimuli [88] mediated by changes in K^+^ channel mRNA in mouse DRGs, with an increase in HCN1 subunit and a down regulation of TREK1, TREK2 (TWIK-Related K^+^ Channel), TRAAK (TWIK-related arachidonic acid-stimulated K^+^ channel), and K_V_1.1 in nociceptors [89,90]. Resta et al. [91] recently showed an HCN current gain of function, a blockade of HCN2 expression, and an upregulation of the HCN regulatory β-subunit MirP1 in DRG neurons from oxaliplatin-treated rats. Mice treated with the HCN blocker ivabradine, which is a nonselective blocker of all four HCN channels [92], abolished the oxaliplatin-induced hypersensitivity to cold and mechanical hyperalgesia [82,89]. Studies in DRG neurons of paclitaxel-treated animals showed changes in the expression of ion channels including the upregulation of K_V_1.2, K_V_11.3, K_ir_3.1, and HCN1 channels along with reductions in K_ir_1.1, K_ir_3.4, and K_2P_1.1 channels [93]. These changes in the ion channels’ expression are accompanied by the increased excitability of nociceptors [88]. HCN1 channels expression increasingly appears to be a common feature in different antineoplastics, suggesting that HCN1 down regulation may be a therapeutic approach to prevent and/or treat CPIN [88].

Given the importance of HCN channels in the pain process [31,77,78,79,80,82,84,94], the reported 3D-structure of the closed (i.e., depolarized) HCN1 subunit has provided new insights on how HCN channels can be modulated for pain treatment, and additionally, address the rational design of new highly selective blockers for HCN1 or HCN2 over HCN4 channels, to avoid depressive effects on heart rate.

## 5. HCN Channels as a Pharmacological Target for Analgesia

**Figure 2:**
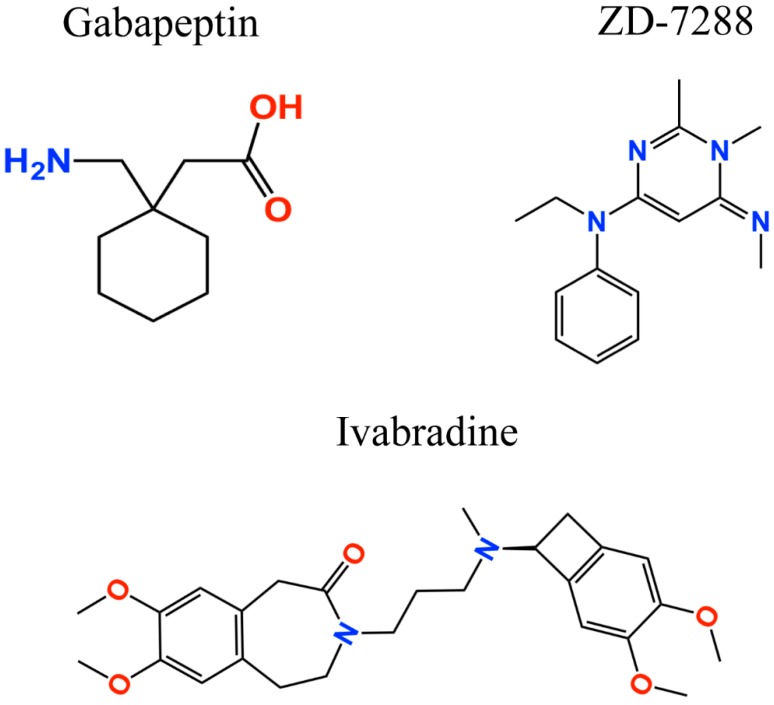
Two-dimensional structures of analgesic drugs targeting HCN channels.

Nociceptive pain involves the transduction, conduction, transmission, modulation, and perception of noxious signals to the brain [95], which are then converted into an electrical signal [96]. The electrical signal is relayed to the dorsal horn, and then to the brain via spinal projections where the information is assessed, and the appropriate response is generated [96,97]. There are compelling evidence supporting the involvement of HCN1–2 subunits in the transmission of electrical signals and the induction of peripheral pain [77,84,98,99,100,101]. Evidence suggests that the inhibition of HCN channels function results in an interruption of electrical signals; therefore, blocking HCN channels can have analgesic effects and reduces pain sensation. For instance, the nonselective HCN channel blocker ZD-7288 (Figure 2) suppresses mechanical and thermal hypersensitivity in different models of neuropathic pain [77,84,98,99,102]. Dysfunction of HCN channel activity is associated with the development and maintenance of chronic pain and inhibition of HCN channel activity produces the anti-nociceptive effect [82,103,104]. Local or systemic administration of ZD-7288 reduced nociceptive behavior in animals with peripheral nerve injury [98]. At the supraspinal level, increased HCN activity appears to be related to chronic pain and comorbidity. For example, HCN1 expression level was increased in the amygdala of rats with chronic constriction of sciatic nerve (CCI) and inhibition of HCN channels was anti-nociceptive [104]. Increased HCN protein expression level and enhanced I_h_ current were also observed in the periaquaductal gray of CCI rats, whereas infusion of ZD-7288 into this brain region attenuated neuropathic pain [52,105]. Moreover, microinfusion of ZD-7288 into the medial prefrontal cortex or the anterior cingulate cortex also produced the anti-nociceptive effect in mice with spared nerve injury [106] or CCI [51]. ZD-7288 infusion into the ventral posterolateral (VPL) nucleus of the thalamus in rats with neuropathic pain or monoarthritis attenuated mechanical allodynia and thermal hyperalgesia in rats with chronic pain [38]. Thus, these results support that the main effect of blockers of HCN channels is on the HCN channels expressed in the peripheral nervous system (PNS). However, there is also evidence that the infusion of HCN blockers in the central nervous system also generates anti-nociceptive effect.

**Figure 3:**
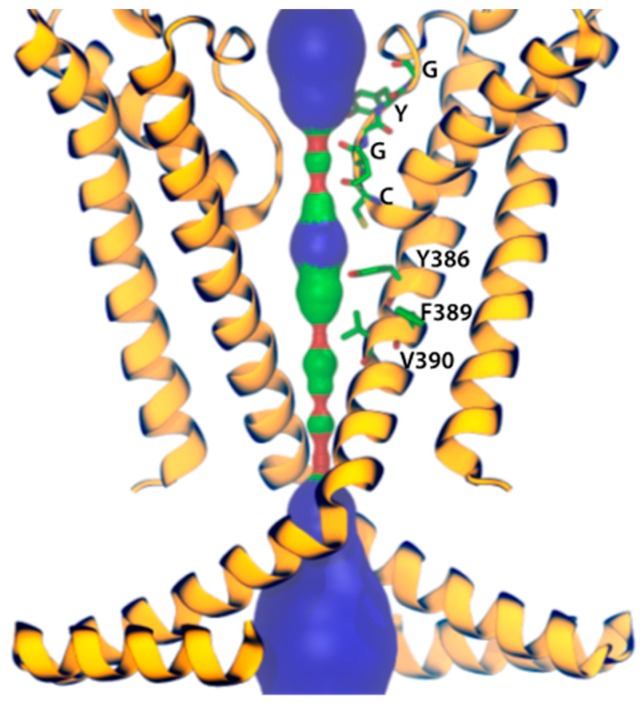
The HCN1 subunit pore. Only two subunits are shown for better visualization. The pore is shown in red where the pore radius is <0.6 Å, green where it is between 0.6 Å and 1.15 Å, and blue where it is >1.15 Å. Residues mediating ivabradine binding and those forming the selectivity filter are represented as sticks.

Additionally, further studies where the HCN channel inhibitors ivabradine and gabapentin were used (the latter is a gold standard for neuropathic pain treatment; Figure 2), corroborated the anti-nociceptive effect [82,107]. Gabapentin, which acts by blocking voltage-gated calcium channels, also showed an upregulated activity of the HCN channels [108]. However, the gabapentin binding mechanism and how it modulates HCN channels remains unclear. Recently, Tae et al. [109] showed that gabapentin reduces HCN4 channel-mediated currents through a hyperpolarized shift in the voltage of activation, with minimal changes for HCN1 channels and none for HCN2 channels. Ivabradine, a nonselective HCN channel blocker with an IC_50_ of ~4 µM for HCN1, HCN2, and HCN4 subunits [110], interacts with the HCN4 subunit pore, binding to amino acid residues Y506, F509, and I510, which stabilize interactions between ivabradine and the HCN4 subunit [111]. On the other hand, ivabradine could interact with the HCN1 subunit pore, binding to amino acid residues Y386, F389, and V390, according to the HCN channel sequence alignment reported by Lee and MacKinnon [4] (Figure 3). A close binding site inspection in the HCN1 structure allows the location of Y386, F389, and V390 residues in the inner cavity, where Y386 and V390 are facing the pore. Further studies are needed to understand the drugs binding to specific amino acid residues, since Y386, F389, and V390 are conserved as members of the voltage-gated pore loop channel superfamily.

Other inhibitors have a greater affinity for specific HCN channels over others. The structural analogs related to zatebradine EC18 and MEL57A displayed selectivity for homomeric HCN channel isoforms [112,113]. Among the isoform-selective phenylalkylamines, EC18 is 6-fold more selective for the HCN4 subunit than the HCN1 subunit [113], and MEL57A is 170- and 30-fold more selective for the HCN1 subunit than the HCN4 and HCN2 subunits, respectively [112]. Furthermore, several selective blockers of the HCN1 channel, 2,2-di-substituted indane derivatives, with 10-fold selectivity for HCN1 over HCN4 have been reported [114]. Among them, the activity of compound **12m**, which showed in vitro selectivity (IC_50_ HCN1 = 6.4 μM, IC_50_ HCN4 = 5.4 μM), was tested in a spared nerve injury (SNI) model [114]. The contribution of the HCN1 channel to induced allodynia and a selective HCN1 blocker, compound **12m**, for treatment of neuropathic pain were displayed [114].

Amino acid residues involved in the binding of drugs to HCN channels may also be different. Unlike ivabradine, which is predicted to bind to amino acid residues Y386, F389, and V390 of the HCN1 subunit, ZD-7288 binds to residues A425 and I432 located in the S6 transmembrane domain of the HCN2 subunit [115]. Nevertheless, the HCN1 3D-structure (PDB ID: 5U6O) had an enormous impact in understanding structure-function relationships for these channels. Despite the fact it has not bound ligand(s), it could improve our comprehension regarding potential drugs binding sites at the HCN channels pore because the structure might help to localize in a 3D framework the residue(s) which, when mutated, alter the binding of HCN blockers. However, to unravel the complex mechanisms of actions of blockers, the combination of different multidisciplinary strategies is required, as well as the obtaining of new HCN structures in different conformational states with and without ligands. This structural knowledge could lead to improving future computational studies using methods such as docking and molecular dynamics simulations, because so far most of theoretical studies that have been done regarding HCN-drug binding mechanisms were done using models of those channels [111,116,117]. Improved computational studies will give structural insights to development of new HCN modulators (activators and inhibitors), which may be useful for the treatment of several pathologies such as arrhythmias, Parkinson’s disease, and epilepsy [44,118,119,120,121,122,123]. Over the last years, the synergy between both experimental and theoretical approaches to study protein-drug interactions have proved to be successful when used to design new drugs targeting ion channels, as well as to study their binding mechanisms at an atomistic and molecular level [124,125,126].

**Figure 4:**
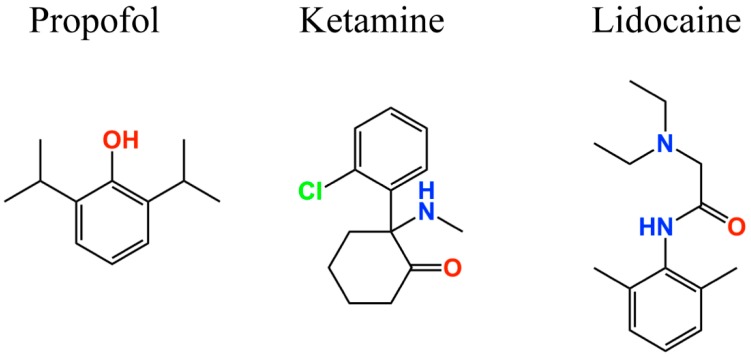
Two-dimensional structures of anesthetic drugs targeting HCN channels.

Regarding anesthetic drugs, there is compelling evidence supporting the involvement of ion channels and receptors, such as type A gamma-aminobutyric acid, *N*-methyl-d-aspartic acid, glycine receptors, and K_2P_ and HCN channels [110,127,128]. Anesthetic drugs induce neuronal hyperpolarization of the membrane potential, thus decreasing the excitability in the central nervous system and causing consciousness status. This hyperpolarization is generated mainly by the activation of K_2P_ channels as well as gamma-aminobutyric acid (GABA), NMDA and glycine receptors. On the other hand, a wide range of volatile anesthetics (e.g., enflurane, isoflurane, halothane [129,130,131,132,133], and xenon [134]), intravenous (e.g., pentobarbital [135], ketamine [136,137], and propofol [45,47,138]) agents, and adjuncts including loperamide (μ-opiate receptor agonist) [139,140] and α2-receptor agonists (e.g., clonidine and dexmedetomidine [141,142,143]) inhibit HCN channel activity [144], and thereby prevent membrane depolarization. The general anesthetic propofol (2,6-di-isopropylphenol) (Figure 4) selectively inhibits HCN1 channels versus HCN2, 3, and 4 [45,138]. Consequently, Tibbs et al. [103] hypothesized that propofol, and congeners, should be antihyperalgesic. Analogs of propofol (alkyl-substituted) present different levels of potency regarding HCN1 inhibition, GABA_A_ receptor (GABA_A_-R) potentiation, and general anesthesia. Thus, 2,6- and 2,4-di-*tert*-butylphenol (2,6- and 2,4-DTBP, respectively) were shown to be more potent HCN1 antagonists than propofol while 2,6- and 2,4-di-*sec*-butylphenol (2,6- and 2,4-DSBP, respectively) were less potent [103]. In contrast, DSBPs (di-*sec*-butylphenols), but not DTBPs (di-*tert*-butylphenols), enhance GABA_A_ receptor activity and are general anesthetics. Also, in a neuropathic pain model, 2,6-DTBP and subhypnotic propofol were antihyperalgesic [103]. These findings are consistent with the fact that alkylphenols are acting through a pathway non-associated with the GABA_A_ receptor, and suggest that central HCN1 channel antagonism may be of limited importance to general anesthesia [103]. Alkylphenols act directly on HCN channels, and have a small effect modifying the lipid bilayer at therapeutic concentrations, despite its hydrophobic characteristic [103]. Thus, the alkylphenol antihyperalgesic target may be the HCN1 channels in the damaged PNS [103].

The volatile anesthetics (i.e., enflurane, halothane, and isoflurane) have previously been shown to inhibit the I_h_ current as well as the current generated by HCN1 and HCN2 channels heterologously expressed [129,130,131,132,133]. Zhou et al. [145] tested the immobilizing, hypnotic, and amnestic effects of isoflurane and sevoflurane in HCN1^−/−^ mice and HCN1^f/f,cre^ mice (with a HCN1 deletion restricted to the forebrain). These tests confirm a role for volatile anesthetic-mediated inhibition of HCN1 when contributing to hypnosis, and likely amnesia but not immobility [144].

For instance, the intravenous anesthetic drug propofol (Figure 4) inhibits I_h_ in hippocampal pyramidal neurons and cortical pyramidal neurons [45] by blocking the HCN1 [138] subunit. Using a heterologous model of recombinant HCN2 and HCN4 subunit proteins, Cacheaux et al. reported that propofol can also block both subunits at clinically relevant concentrations (<10 µM). These findings are supported by results from HCN1 knockout mice, which were less sensitive to the propofol effects [133].

Ketamine, a drug with anesthetic, analgesic, and psychotropic effects, also inhibits the HCN1 subunit in cortical pyramidal neurons (Figure 4), and its effects are attenuated in HCN1 knockout mice [136,146]. On the other hand, the local anesthetic lidocaine (Figure 4) inhibits HCN1, HCN2, and HCN4 subunits, as well as heteromeric HCN1–HCN2 channels acting in a dose-dependent manner over a concentration range relevant for systemic use [147]. However, propofol, ketamine, and lidocaine action mechanisms are not completely understood due to the lack of information about the amino acids that integrate their binding site. The HCN1 three-dimensional structure provides new insights regarding how HCN channels can be inhibited to treat pain, as well as information regarding their biophysical properties [124,125].

Volatile anesthetic drugs, such as the commonly used isoflurane and halothane, decrease I_h_ in cortical neurons mediated by monomeric and heteromeric HCN2 and HCN1 channels [132,133]. Isoflurane effects have been studied in the HCN1 subunit. The HCN1 knockout revealed that isoflurane targets the HCN1 subunit by reducing I_h_ in motoneurons and cortical pyramidal neurons [133]. In these neurons, from HCN1 knockout mice, electrical changes induced by the isoflurane anesthetic were absent, verifying the HCN1 subunit specific contribution to isoflurane effects [133].

## 6. Challenges and Future Directions in Structure-Based Drug Design Targeting HCN Channels

Rational drug design targeting ion channels with specific properties is a constantly evolving research field. The design offers revolutionary advantages for the development of new therapeutic drugs through the use of newly developed methods such as computational tools and structure-based approaches, where the structural, physical-chemical, and pharmacological properties of the ligands and targets are critical to gain high potency and binding affinity.

Nowadays, for drugs whose molecular target is an ion channel with a known 3D structure, experimental and theoretical approaches are used to study protein-ligand interactions. Structure-based drug design (SBDD) is most powerful when it is a part of an entire drug discovery process. This is a handy and versatile computational tool that examine the 3D structure of a given target (previously obtained by X-ray crystallography, nuclear magnetic resonance (NMR) spectroscopy, cryoelectron microscopy, or any other molecular modeling technique [148]), followed by the study of binding properties and affinity of a given set of ligands for their molecular target [125]. This approach, coupled with combinatorial chemistry may lead to the parallel synthesis of focused compound libraries, and has been particularly helpful for the identification of hits and lead compounds, targeting membrane proteins in cases where traditional methods have failed due to the target’s nature [149]. It is also important to be aware that the SBDD guides the discovery of a drug lead, which is not a drug product, but, especially, a molecule with affinity for a target in at least the micromolar range [150]. Additionally, the crosstalk between different research areas such as medical chemistry, biochemistry, bioinformatics, genomics, proteomics, and metabolomics has contributed to the development of new computational tools for the rational design of ion channel modulators [125]. Thus, the use of established methods (i.e., docking, virtual screening, de novo drug design, and molecular simulations involving electrophysiological approximations [151]) will radically change the way in which new modulators targeting ion channels with higher potency and affinity are designed. In this way, the reported three-dimensional structure of the HCN1 subunit will provide new insights on how HCN channels can be modulated for pain treatment.

## 7. Conclusions

The cDNA cloning and partial characterization of different HCN subunits have set the pace for their extensive study using biochemical, biophysical, genetic, and cellular approaches. HCN subunits are highly regulated proteins which respond to different stimuli.

Hyperpolarization-activated cyclic nucleotide-gated channels are widely distributed in both excitable (and non-excitable) cells of the nervous system and the heart. Their expression patterns in different tissue and cells are affected by different pathological conditions. These channels transmit electrical signals in excitable cells such as neurons; for instance, they control heart rate and also inhibit pain [97,110]. Despite the involvement of HCN channels in various diseases [44], it is unclear how they can be pharmacologically targeted to alleviate illness symptoms such as pain. Hyperpolarization-activated cyclic nucleotide-gated channels are overexpressed in inflammatory and neuropathic pain, and HCN blockers have been shown to reduce neuronal excitability and to ameliorate painful states in animal models. However, HCN channels are critical in cardiac action potential, and HCN blockers used so far in pre-clinical models (e.g., ZD-7288 and ivabradine) do not discriminate between cardiac and non-cardiac HCN isoforms, generating bradycardia by the inhibition of HCN4-dependent pacemaking activity in the heart [82,107].

Therefore, the 3D structure of the human HCN1 subunit [4] has opened new possibilities for the rational design of highly-selective HCN modulators, especially those handy to treat pain. Based on the HCN1 structure, it is interesting to speculate about the structure and functional relationship associated with HCN blockers. Further functional studies coupled with mutagenesis experiments as well as computational studies are needed to identify the key amino acids that integrate drug binding sites in HCN channels.
